# Health indices for the evaluation and monitoring of health in children and adolescents in prevention and health promotion: a scoping review

**DOI:** 10.1186/s12889-021-12335-x

**Published:** 2021-12-20

**Authors:** Albulena Selmani, Michaela Coenen, Stephan Voss, Caroline Jung-Sievers

**Affiliations:** 1 Institute for Medical Information Processing, Biometry and Epidemiology-IBE, Chair of Public Health and Health Services Research, LMU Munich, Munich, Germany; 2 Pettenkofer School of Public Health, Munich, Germany

**Keywords:** Health index, Health status indicators, Composite index, Child health, Adolescent health, Evaluation, Monitoring, Surveillance

## Abstract

**Background:**

Health indicators are used in different settings to monitor health outcomes. Child and adolescent health is arguably one of the most important areas for the application of indices and indicators in prevention and health promotion. Although single health indicators may be better suited to display the complexity of the health status and its determinants, a selected set of indicators will still offer a complex picture. Therefore, it is argued that a group of indicators combined into an index may offer a pragmatic tool that is easier to use in order to inform stakeholders.

**Methods:**

A scoping review was conducted to identify and describe health indices that monitor and evaluate health of children and adolescents and to appraise the quality and value of the identified indices that may guide the further applications of these indices in particular settings. The three bibliographic databases MEDLINE, EMBASE and PsycINFO were searched and a double screening of titles and abstracts as well as double screening of full texts was performed. Indices contained in these studies were analysed in terms of focus and composition and evaluated in terms of quality criteria.

**Results:**

The scoping review identified 36 eligible studies with 18 health indices in six thematic categories. Of the identified indices, seven indices focus on anthropometrical variables, three indices focus on special aspects of newborns and five indices focus on oral health. One index assesses “healthy lifestyle” and one “functional ability” whereas one index a combination of different aspects. Most indices are calculated by using primary health data.

**Conclusions:**

Alone or in combination with single sets of indicators, indices in six major thematic domains may be used as pragmatic tools for monitoring children’s and adolescents´ health and the evaluation of interventions in health promotion and prevention settings.

**Supplementary Information:**

The online version contains supplementary material available at 10.1186/s12889-021-12335-x.

## Background

Health indicators are used in different settings to monitor health [1]. For example, the World Health Organization (WHO) published a global reference list of 100 core health indicators and health-related SDGs (Sustainable Development Goals) [2]. These are standard sets of core indicators prioritized by the global community to provide concise information on the health situation and trends, including responses at national and global levels. Its purpose is to serve as a normative guidance for the selection of standard indicators and their definitions that countries and partners can use for monitoring health in accordance with their respective health priorities and capacity. The Organisation for Economic Co-operation and Development (OECD) compares key indicators and reports on trends over time on population health, risk factors for health and health system performance across OECD members and partner countries [3].

Child and adolescent health is arguably one of the most important areas for the application of indices and indicators in prevention and health promotion [4]. Investment in child and adolescent health and control of adverse determinants will have a long-lasting effect on the whole population, including influencing the health of the next generation [4]. Furthermore, young people are citizens in their own right, yet largely unable to act as self-advocates, particularly at the population level [5]. Children, particularly in infancy, are unable to demand better services for themselves, and therefore information systems such as indicators act as the child’s and adolescent’s advocate [4].

The World Health Organization describes health as a “state of complete physical, mental and social well-being and not merely the absence of disease or infirmity” [6]. Understanding health as a complex and multidimensional phenomenon, indicators from a wide range of domains come into consideration to evaluate health.

Although a combination of single health indicators may help to understand the complexity of the health status of children, a selected set of indicators may be overcomplex, not least by their sheer numbers or unclear selection or priority setting algorithm [1]. One approach to overcome this complexity and to gain feasibility is to combine indicators into a composite score or index [1]. An index is argued to be more accessible for advocacy and political intervention decisions [1]. Furthermore, a well-designed index may provide a comprehensive vision of a multidimensional phenomenon, to “allow for the setting of national benchmarks and for further international comparisons and is a starting point for analysis and discussion” [1].

However, up to date there is no international consensus on the selection and use of indices despite many narrative reviews, opinions and policy briefs [1, 7]. Therefore, our objectives were to scope the existing evidence with the two specific aims: a) to identify and describe health indices that monitor and evaluate health of children and adolescents, and b) to appraise the quality and value of the identified indices that may guide the further applications of these indices in particular settings.

## Methods

This scoping review was conducted according to the PRISMA Extension for Scoping Reviews (PRISMA-ScR) [8] and performed according to a preregistered protocol published in the open science framework under the following link: https://osf.io/htgeu.

### Search methods for the identification of publications

The three bibliographic databases MEDLINE, EMBASE and PsycINFO were searched using the following search terms and truncations for a) the population: child, children, teenager*, youth, adolescen*; b) the interest: index*, indices, indicator*; c) the context: health promotion, prevention, monitoring, reporting, public health surveillance, population surveillance. The search strategy was adapted for each database. Database searches were conducted on May 26, 2020 and limited to studies published in English and German. No restriction based on the year of publication was applied. The search strategy was designed by the first author and refined in team discussions with all coauthors. The search strategies for the three databases can be found in Supplementary file 1. Additionally, the references of included reviews were searched for eligible records.

### Eligibility criteria

We applied the following inclusion and exclusion criteria. Criteria were reported according to the PICo (Population, Interest, Context) scheme.

#### Inclusion criteria

Population:

- Children/adolescents aged 0–19 years, including 19 year olds (according to WHO classifications of adolescence up to 19 years [9])
- Mothers with their children
- Children, adolescents (aged 0–19 years) and their parents or family

Interest:

- Health indices combining different health indicators into a composite score

Context:

- Health promotion and prevention
- Monitoring of health indicators
- Oral health

#### Exclusion criteria

Population:

- Aged over 19 years
- General population or clinical population

Interest:

- Indicators from areas other than health
- Indicators on Quality of Life
- Studies with the purpose of examining quality criteria of a diagnostic instrument

Context:

- Clinical interventions or clinical settings
- Indicators with a clinical aim such as health care delivery and quality of care
- Association studies and predictors of health indicators

### Study selection

The search results were exported to EndNote and duplicates were removed. All four authors screened the titles, abstracts and full texts of the publications in a stepwise procedure. Firstly, for title and abstract screening the web-based application rayyan was used. All hits were independently screened by the first author and a second coauthor. Any disagreements were resolved through discussion between the two reviewers and in case of disagreement with a third reviewer. Secondly, for the full text screening, the first author screened all publications while 30% of the publications were independently screened by one of the other authors. Disagreements on study selection and data extraction were solved by consensus and discussion with other reviewers if needed.

### Identification of indices

All eligible studies were searched for indices defined as combining different indicators into a composite score. The definition of an index was based on the definition of the OECD for ´composite Indicator´ [10] and Ashraf et al. [11].

### Data extraction, management and synthesis

A customized Excel-based data extraction form was developed. The data chart captured the relevant information on key study characteristics such as authors, year of publication, study type, country, setting, population, number of participants and indices reported. For the data extraction, the first author independently charted data from all eligible articles with 30% of the data checked and extracted by a second author.

### Quality appraisal of indices

The indices were assessed in terms of five quality domains: index is 1) valid, 2) consistent, 3) sensitive, 4) feasible and 5) defined (see Table 3). These criteria are based on quality criteria used in the Child Health Indicators of Life and Development (CHILD) project [5], which is part of the European Community Health Monitoring Programme (HMP) and are described in the following:

- Validity: Validity is divided into two criteria. Face validity pertains to the “ability of the indicator to measure what it says it measures” [5]. Construct validity “means that the indicator demonstrates an expected empirical relationship with other related indicators” [5].
- Consistency: Consistency is defined as “having reliability in measurement, so that variation in values is true variation not random error” [5].
- Sensitivity: Sensitivity examines whether the index is appropriate to assess change over time [5].
- Feasibility: Feasibility means that “reliable source data must be available” [5].
- Defined: An index is defined, when it is “unambiguous in its data construct” [5].

For each of the criteria validity, consistency and sensitivity, the reviewer’s assessment was given in a first column. Additionally, it was indicated how the assessment was reported in the included studies. For this judgement, further literature was consultated in case that no information could be retrieved from the study itself. It is further indicated whether the data collected to calculate the index is available from routine data, or whether primary studies were necessary to collect the data. For the criterion defined, it was assessed whether there is a clear guideline, such as a formula for calculating the index. Further literature was enquired if the information was not contained in the studies per se.

## Results

### Selection of sources of evidence

**Fig. 1:**
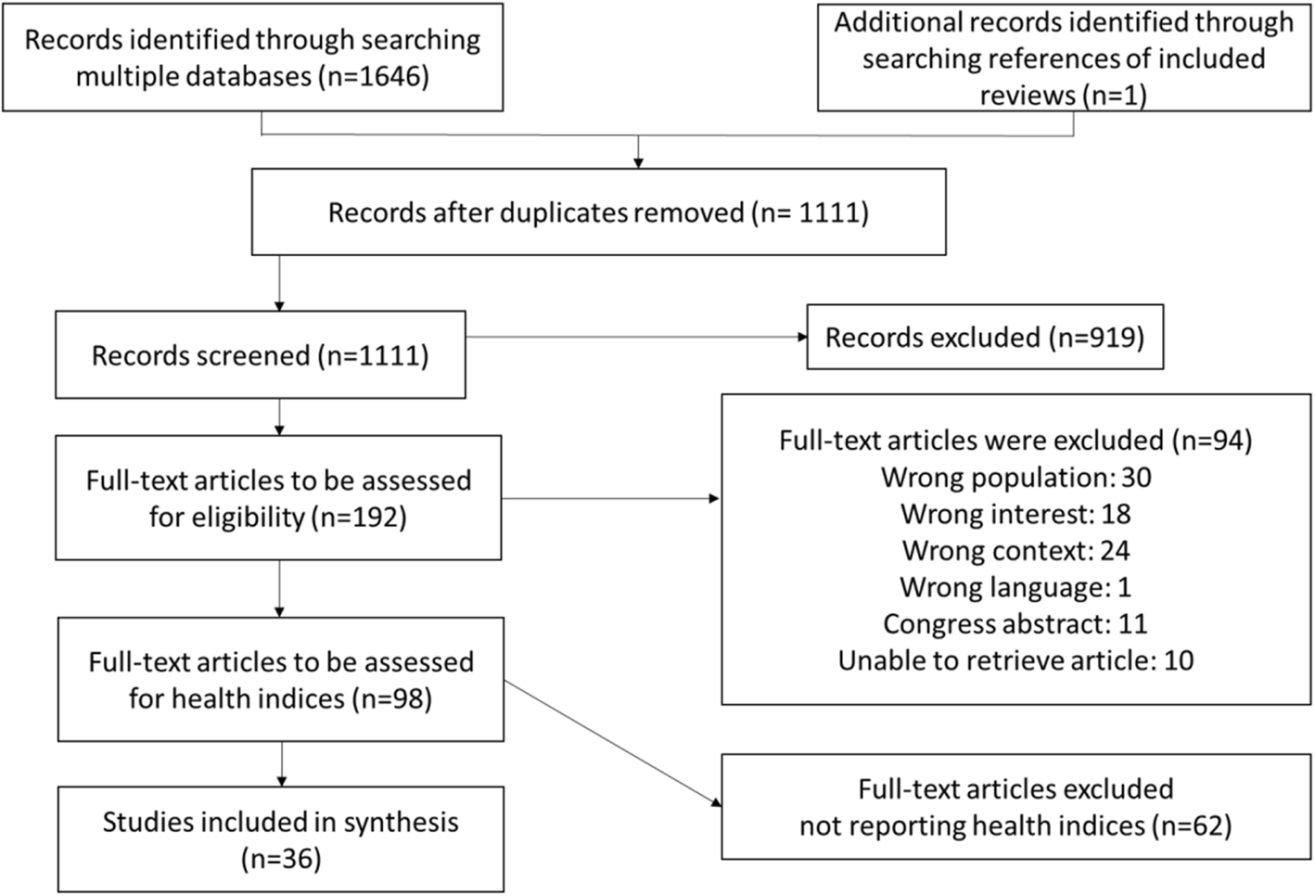
PRISMA flow chart of studies identified, screened and included

The selection of sources of evidence is shown in a PRISMA flow chart (Fig. 1) [8]. In total, 1646 records were identified by searching the databases MEDLINE, EMBASE and PsycINFO. One additional eligible record was identified through searching the references of a review. After screening titles, abstracts and full texts, 98 articles remained with 36 studies finally eligible.

### Characteristics of sources of evidence

Table 1 shows the characteristics of studies with references and year of publication. The included studies were published from 1980 to 2019 and comprised study designs such as observational studies (*n* = 20), interventional studies (*n* = 8) and reviews (*n* = 8). Stratifying studies by WHO regions [12], most of the identified studies are from the Region of the Americas (*n* = 15; 41.7%), followed by the European Region (*n* = 11; 30.6%), Eastern Mediterranean Region (*n* = 4; 11.1%) and Western Pacific Region (*n* = 2; 5.6%). The most frequently reported context was school settings. The age of the populations in the studies ranged from 0 to 19 years. The number of persons per study ranges from 64 to 16,192. In each study, between one to four indices were reported.

**Table 1:** Identified studies

Authors	Year of publication	Study type	Country^a^	Setting	Population age (years)^b^	Indices in this paper
Abalkhail & Shawky [13]	2002	Observational	Saudi Arabia	Schools	10–19	BMI percentiles for age and sex, Mid-arm muscle circumference (MAMC)
AlBuhairan et al. [14]	2015	Observational	Saudi Arabia	Schools	10–19	Body Mass Index (BMI), BMI percentiles for age and sex
Antunes et al. [15]	2004	Observational	Brazil	n.a.^c^	11–12	Decayed, missing and filled teeth (DMFT), Significant Caries Index (SiC index)
Ardic & Erdogan [16]	2017	Interventional	Turkey	Schools	12–15	Body Mass Index (BMI)
Austin et al. [17]	2007	Interventional	USA	Schools	Grade 6–7	Body Mass Index (BMI), BMI percentiles for age and sex
Ayatollahi & Bagheri [18]	2010	Observational	Iran	School	6.5–11.5	Body Mass Index (BMI)
Bay et al. [19]	2017	Review	n.a.	Schools	n.a.	Body Mass Index (BMI), Waist-to-height ratio
Beauchamp et al. [20]	2011	Interventional	Canada	Schools	Mean age 14.07	Body Mass Index (BMI), BMI percentiles for age and sex, Waist-to-hip ratio
Biscaglia et al. [21]	2019	Observational	Jordan, Lebanon, Syria, Gaza Strip and West Bank	Schools	12	Decayed, missing and filled surfaces (DMFS), Decayed, missing and filled teeth (DMFT), Significant Caries Index (SiC index)
Detty [22]	2013	Observational	USA	Schools	Grade 3	Body Mass Index (BMI)
Do & Spencer [23]	2007	Observational	Australia	n.r.^d^	8–13	Decayed, missing and filled surfaces (DMFS)
Elder et al. [24]	2014	Interventional	USA	Recreation centres	5–8	Body Mass Index (BMI), BMI percentiles for age and sex, BMI z-scores for age and sex
Flynn et al. [25]	2006	Review	n.a.	n.a.	n.a.	Body Mass Index (BMI), BMI percentiles for age and sex
Gardner et al. [26]	2016	Observational	Australia	n.a.	0–16	Body Mass Index (BMI)
Gissler et al. [27]	1992	Observational	Finland	n.a.	0–7	Ponderal index, 1-min Apgar score, 5-min Apgar score, perinatal health index
Greer et al. [28]	2003	Observational	USA	Schools	5–9	Decayed, missing and filled teeth (DMFT)
Hebestreit [29]	2019	Review	Europe	n.a.	n.a.	Body Mass Index (BMI)
Kilanowski & Lin [30]	2014	Interventional	USA	Schools	11–17	Body Mass Index (BMI), BMI percentiles for age and sex
Kilanowski & Ryan-Wenger [31]	2007	Observational	USA	n.a.	0–12	BMI percentiles for age and sex, decayed, missing and filled teeth (DMFT)
Kohen et al. [32]	2007	Observational	Canada	Schools	4–11	Health Utilities Index (HUI)
Köhler [33]	2006	Review	Sweden	n.a.	0–19	Health indicators for Swedish children
Köhler [1]	2016	Review	n.a.	n.a.	n.a.	Health indicators for Swedish children
Koivusilta et al. [34]	2006	Observational	Finland	n.a.	12–16	Body Mass Index (BMI), BMI percentiles for age and sex
Kulaga et al. [35]	2010	Observational	Poland	Schools	6.5–18.5	Body Mass Index (BMI), BMI z-scores for age and sex
Kuntz & Lampert [36]	2013	Observational	Germany	n.a.	11–17	Body Mass Index (BMI), BMI percentiles for age and sex, Healthy Lifestyle Index (HLI)
Muratbegovic et al. [37]	2010	Observational	Bosnia and Herzegovina	Communities	6	Decayed, missing and filled teeth (DMFT)
Nyström et al. [38]	2016	Observational	Sweden	n.r.	0–18	Body Mass Index (BMI)
Ottolenghi et al. [39]	2007	Review	Europe	n.a.	n.a.	Decayed, missing and filled teeth (DMFT)
Peoples-Sheps et al. [40]	1998	Review	USA	n.a.	1–19	Body Mass Index (BMI), BMI percentiles for age and sex
Smadi [41]	2017	Observational	Jordan	Schools	12–18	Decayed, missing and filled teeth (DMFT), Significant Caries Index (SiC index)
Stamm [42]	1980	Review	Canada, USA	n.a.	n.a.	Decayed, missing and filled teeth (DMFT), deft index (for primary/baby teeth)
Stamm et al. [43]	1980	Observational	Canada	n.a.	13–14	Decayed, missing and filled teeth (DMFT), Oral Hygiene Index (simplified) OHI(S)
Tyler [44]	2004	Observational	USA	School based health centre	7–12	Body Mass Index (BMI), BMI percentiles for age and sex
Vogeltanz-Holm & Holm [45]	2018	Interventional	USA	Schools	Mean age 8.75	Body Mass Index (BMI), BMI percentiles for age and sex, BMI z-scores for age and sex
Whittemore et al. [46]	2013	Interventional	USA	Schools	14–17	Body Mass Index (BMI), BMI percentiles for age and sex
Wrobel & Mielnik-Blaszczak [47]	2012	Interventional	Poland	Schools	12–13	Decayed, missing and filled surfaces (DMFS)

^a^If there were no data on country but regions or continents, this information was noted in this column

^b^If there was no information on population age but on mean age or school grade, this information was noted in this column

^c^n.a. indicates that the information was not applicable

^d^n.r. indicates that the information was not reported

### Health indices with quality assessment

Table 2 shows 18 identified indices with respective quality assessments in Table 3. Each index is assigned to a category that describes the overall theme. These thematic categories emerged from the data in a deductive process. In total, six different topic-specific categories for the 18 indices were chosen: namely Category 1: Anthropometrical Indices, Category 2: Special Indices for newborns, Category 3: Indices for oral health, Category 4: Indices on lifestyle, Category 5: Indices on functional ability and Category 6: Indices combining different aspects (such as prenatal, postnatal and behavioural aspects).

**Table 2:** Identified Indices

Categories	Index	Variables included in the index	References^a^
Anthropometrical	Body Mass Index (BMI)	Height, weight	Abalkhail & Shawky, 2002 [13]; AlBuhairan et al. 2015 [14]; Ardic & Erdogan, 2017 [16]; Austin et al., 2007 [17]; Ayatollahi & Bagheri, 2010 [18]; Bay et al., 2017 [19]; Beauchamp et al., 2011 [20]; Detty, 2013 [22]; Elder et al., 2014 [24]; Flynn et al., 2006 [25]; Gardner et al., 2016 [26]; Hebestreit, 2019 [29]; Kilanowski & Lin, 2014 [30]; Koivusilta et al., 2006 [34]; Kulaga et al., 2010 [35]; Kuntz & Lampert, 2013 [36]; Nyström et al., 2016 [38]; Peoples-Sheps et al., 1998 [40]; Tyler, 2004 [44], Vogeltanz-Holm & Holm, 2018 [45]; Whittemore et al.,2013 [46]
Anthropometrical	BMI percentiles for age and sex	Height, weight	Abalkhail & Shawky, 2002 [13]; AlBuhairan et al. 2015 [14]; Austin et al., 2007 [17]; Beauchamp et al., 2011 [20]; Elder et al., 2014 [24]; Flynn et al., 2006 [25]; Kilanowski & Lin, 2014 [30]; Kilanowski & Ryan-Wenger, 2007 [31]; Koivusilta et al., 2006 [34]; Kuntz & Lampert, 2013 [36]; Peoples-Sheps et al., 1998 [40]; Tyler, 2004 [44]; Vogeltanz-Holm & Holm, 2018 [45]; Whittemore et al.,2013 [46]
Anthropometrical	BMI z-scores for age and sex	Height, weight	Elder et al., 2014 [24]; Kulaga et al., 2010 [35]; Vogeltanz-Holm & Holm, 2018 [45]
Anthropometrical	Waist-to-height ratio	Height, waist circumference	Bay et al., 2017 [19]
Anthropometrical	Waist-to-hip ratio	Waist circumference, hip circumference	Beauchamp et al., 2011 [20]
Anthropometrical	Mid-arm muscle circumference (MAMC)	Mid-arm circumference, triceps skin fold thickness	Abalkhail & Shawky, 2002 [13]
Anthropometrical	Ponderal index	Weight, height	Gissler et al., 1999 [27]
Newborns	1-min Apgar score	Appearance (skin colour), pulse (heart rate), grimace response (reflexes), activity (muscle tone), respiration (breathing rate and effort)	Gissler et al., 1999 [27]
Newborns	5-min Apgar score	Appearance (skin colour), pulse (heart rate), grimace response (reflexes), activity (muscle tone), respiration (breathing rate and effort)	Gissler et al., 1999 [27]
Newborns	Perinatal health index	Birthweight, gestational age, 5-min Apgar score, birthweight by gestational age, perinatal diagnosis	Gissler et al., 1999 [27]
Dental	Decayed, missing and filled surfaces (DMFS)	Decayed surfaces (DS), missing surfaces (MS), filled surfaces (FS)	Biscaglia et al., 2019 [21]; Do & Spencer, 2007 [23]; Wrobel & Mielnik-Blaszczak, 2012 [47]
Dental	Decayed, missing and filled teeth (DMFT)	Decayed teeth (DT), missing teeth (MT), filled teeth (FT)	Antunes et al., 2004 [15]; Biscaglia et al., 2019 [21]; Greer et al., 2003 [28]; Kilanowski & Ryan-Wenger, 2007 [31]; Muratbegovic et al., 2010 [37]; Ottolenghi et al., 2007 [39]; Smadi, 2017 [41]; Stamm, 1980 [42]; Stamm et al., 1980 [43]
Dental	Significant Caries Index (SiC index)	Decayed teeth/ surfaces (DT/S), missing teeth/surfaces (MT/S), filled teeth/surfaces (FT/S)	Antunes et al., 2004 [15]; Biscaglia et al., 2019 [21]; Smadi, 2017 [41];
Dental	Deft index (for primary/baby teeth)	Decayed teeth, teeth extracted due to caries, filled teeth	Stamm, 1980 [42]
Dental	Oral Hygiene Index (simplified) OHI(S)	Debris score, calculus score	Stamm et al., 1980 [43]
Lifestyle	Healthy Lifestyle Index (HLI)	Current tobacco consumption, body mass index, physical activity, regular consumption of alcohol, consumption of fresh fruit and vegetables, daily use of electronic media: television/video, computer/internet and game console	Kuntz & Lampert, 2013 [36]
Functional ability	Health Utilities Index (HUI)	Vision, hearing, speech, ambulation, dexterity, cognition, emotion, pain	Kohen et al., 2007 [32]
Combination of different aspects	Health indicators for Swedish children	Children injured by external causes, children exposed to tobacco in the womb, children with low birthweight, children that are breastfed, teenage abortions, children vaccinated against MPR (measles, parotitis and rubella)	Köhler, 2006 [1]; Köhler, 2016 [1]

^a^Reference of the studies identified in this scoping review reporting the index

**Table 3:** Assessment of quality criteria

Index	Face validity^a^	Face validity^b^	Construct validity^a^	Construct validity^b^	Consistency^a^	Consistency^b^	Sensitivity^a^	Sensitivity^b^	Feasibility	Defined
Body Mass Index (BMI)	Yes	Yes [48]	Yes	Yes	Discussed	Yes	Yes	Yes	Primary data	Yes [48]
BMI percentiles for age and sex	Yes	Yes [48]	Yes	Yes	Yes	Yes	Yes	Yes	Primary data	Yes [48]
BMI z-scores for age and sex	Yes	Yes [48]	Yes	Yes	Yes	Yes	Yes	Yes	Primary data	Yes [48]
Waist-to-height ratio	Yes	Yes	Yes	Yes	Yes	Yes [49]	Yes	Yes [49]	Primary data	Yes [50, 51]
Waist-to-hip ratio	Yes	Yes [52]	Yes	Yes	Yes	Yes [52]	Yes	Yes [52]	Primary data	Yes [52]
Mid-arm muscle circumference (MAMC)	Yes	Yes	Yes	Yes	Yes	Yes	n.a.^c^	n.r.^d^	Primary data	Yes [13]
Ponderal index	Yes	Yes [53]	Yes	Yes [53]	Yes	Yes [53]	Yes	Yes [53]	Primary data	Yes [27]
1-min Apgar score	Yes	Yes [54]	n.a.	n.r.	No	No [54]	n.a.	n.r.	Routine data	Yes [54]
5-min Apgar score	Yes	Yes [54]	n.a.	n.r.	No	No [54]	n.a.	n.r.	Routine data	Yes [54]
Perinatal health index	Yes	n.r.	n.a.	n.r.	Discussed	Yes	No	No	Routine data	Yes
Decayed, missing and filled surfaces (DMFS)	Yes	Yes [55]	Yes	Yes	Yes	Yes	Yes	Yes [55]	Primary data, routine data	Yes
Decayed, missing and filled teeth (DMFT)	Yes	Yes [55]	Yes	Yes	Yes	Yes	Yes	Yes [55]	Primary data	Yes
Significant Caries Index (SIC index)	Yes	Yes [55]	Yes	Yes	Yes	Yes	Yes	Yes [55]	Primary data	Yes
Deft index (for primary/baby teeth)	Yes	Yes [55]	Yes	Yes	Yes	Yes	Yes	Yes	Primary data	Yes
Oral Hygiene Index (simplified) OHI(S)	n.a.	n.r.	n.a.	n.r.	Yes	Yes	Yes	Yes	Primary date	Yes
Healthy Lifestyle Index (HLI)	Discussed	Discussed	Yes	Yes	Discussed	n.r.	No	No	Primary data	Yes
Health Utilities Index (HUI)	Yes	Yes [56]	n.a.	n.r.	Discussed	Yes [56]	Yes	Yes [56]	Primary data	Yes
Health indicators for Swedish children	No	No	n.a.	n.r.	Yes	Yes	Yes	Yes	Routine data	Yes

^a^assessment of the reviewer

^b^as described in the identified paper or further literature; references for information from further literature are cited

^c^n.a. indicates that the reviewer could not make a reasoned assessment and statement due to missing information on the quality criterion in the identified studies or further literature

^d^n.r. indicates that the information is not reported

#### Category 1: indices of anthropometrical variables

We identified seven indices that we attributed as anthropometrical: body mass index (BMI) [13, 14, 16–20, 22, 24–26, 29, 30, 34–36, 38, 40, 44–46], BMI percentiles [13, 14, 17, 20, 24, 25, 30, 31, 34, 36, 40, 44–46], BMI z-scores [24, 35, 45], waist-to-height ratio [19], waist-to-hip ratio [20], mid-arm muscle circumference [13] and ponderal index [27].

The first three indices shown in Table 2 are the Body Mass Index (BMI), BMI percentiles for age and sex and BMI z-scores. The BMI is a measure for indicating nutritional status [48]. It is defined as weight in kilograms divided by the square of the height in metres [48]. BMI is also recommended for use in children and adolescents where the BMI is calculated and then compared with z-scores or percentiles [48]. Since the ratio between weight and height varies with sex and age during childhood and adolescence, the cut-off values that determine the nutritional status of those aged 0–19 years are gender and age-specific [48]. In the definition of the WHO, “the cut-off points of the 2006 BMI-for-age reference for children aged 0–5 years for the diagnosis of overweight and obesity were set as the 97th and the 99th percentile, respectively. For those aged 5–19 years, overweight is defined as a BMI-for-age value over +1 SD and obesity as a BMI-for-age value over +2 SD.” [48]. According to the identified studies and the WHO [48], the three indices provide face and construct validity, consistency and sensitivity. Regarding the consistency criteria, there are in fact standardized measuring procedures and this argument is used to justify consistency of the data [36], but this is not guaranteed if weight and height are self-reported by participants like in the study of Koivusilta et al. [34].

The waist-to-height ratio [19] is the waist circumference divided by the height in centimetres [50]. It is used for obesity assessment with the rationale that, for a given height, there is an acceptable degree of fat in the upper portion of the body [51]. It fulfils the quality criteria validity, consistency and sensitivity [49].

The waist-to-hip ratio is the waist circumference divided by the hip circumference [52]. It was suggested as an measure of body fat distribution and can be measured more precisely than skin folds [52]. After consulting further literature [52] on face validity, consistency and sensitivity, it can be stated that waist-to-hip ratio also meets most quality criteria.

The mid-arm muscle circumference (MAMC) is calculated using the mid-arm circumference and the triceps skin fold thickness and is used for the assessment of muscle content [13]. It is described as valid and consistent in the identified study [13], but lacks information on sensitivity.

The ponderal index is calculated as weight/lenght^3^ and is used to measure newborns´ health [27]. It is a valid, consistent and sensitive measure [53].

#### Category 2: special indices for newborns

We identified three indices that we attributed as indices for newborns: The 1-min Apgar score [27], the 5-min Apgar score [27] and the perinatal health index [27].

The Apgar score is a rapid method for assessment immediately after birth and in response to resuscitation [54]. It includes information on skin colour, heart rate, reflexes, muscle tone and respiration [54]. Each category is scored 0, 1, or 2; the meaning of the scorings is defined for each category [54]. “The score is recorded at 1 min and 5 min in all infants with expanded recording at 5-min intervals for infants who score 7 or less at 5 min, and in those requiring resuscitation as a method for monitoring response. Scores of 7 to 10 are considered reassuring. “ [54]. Referring to other literature [54], face validity is given, whereas consistency is not due to its high subjectivity and inter-rater variability [54].

The perinatal health index was developed by Gissler et al. [27] to investigate how well children’s health can be predicted by perinatal outcomes. The index is calculated using information on birthweight, gestational age, 5-min Apgar score, birthweight by gestational age and perinatal diagnoses [27]. Each of the five categories is assessed and given a score [27]. The combined value of the five categories builds the final perinatal health index [27]. The index classifies the newborns studied into three categories: a healthy newborn; a newborn with some perinatal problem(s) which do not necessarily affect the subsequent health; a newborn with severe perinatal problems causing a major risk for subsequent health [27]. The perinatal health index is reported to not be a sensitive index [27]. It is stated to be a consistent index, however, with the limitation that the Apgar score is included in this index as discussed above [27].

#### Category 3: indices for oral health

We identified five indices that we attributed as dental: DMFS [21, 23, 47], DMFT [15, 21, 28, 31, 37, 39, 41–43], Significant Caries Index (SiC index) [15, 21, 41], deft index [42] and the simplified Oral Hygiene Index (OHI(S)) [43].

The DMFS includes information on decayed, missing and filled surfaces [21, 23, 47] whereas the DMFT includes information on decayed, missing and filled teeth [15, 21, 28, 31, 37, 39, 41–43]. These indices indicate the number of teeth/surfaces that were affected by caries, filled or missing as a result of caries and are therefore used to assess dental caries [21]. The SiC index is defined as the mean of DMFT or DMFS for one-third of the study group with the highest caries scores [15, 21, 41]. The deft index includes the variables decayed teeth, teeth extracted due to caries and filled teeth and is used for the caries assessment of primary/baby teeth [42]. The OHI(S) is used for oral hygiene assessment by examining the presence of plaque on the surface of the teeth witch is expressed in the two indicators debris and calculus score [43].

Of the five indices reporting on oral health, four met the quality criteria on validity, consistency and sensitivity. These are DMFT, DMFS, SiC index and deft index. Face validity and sensitivity were reported as good in published WHO literature on oral health surveys [55]. The OHI(S) is stated to be consistent and sensitive [43].

#### Category 4: index on lifestyle

We identified one index that focuses on lifestyle, the Healthy Lifestyle Index (HLI) [36]. The HLI is based on data regarding smoking behaviour, body mass index, physical activity, use of electronic media, alcohol intake and fruit and vegetable consumption [36]. The values of the six individual indicator constructs are assessed in a dichotomised way (0 = negative, 1 = positive) and then added into a composite score [36]. The index is described as being not sensitive, the construct validity is described as good whereas the face validity is under discussion [36]. As the HLI examines health-related behaviour, it is argued that at least some of the most important behaviour-based factors influencing health in adolescence are included [36]. Yet individual health behaviours, even when taken together, are not capable of fully reflecting a health-related lifestyle, since factors such as resources, orientations, attitudes, opinions and knowledge may also be important to be considered [36]. Regarding consistency, it should be noted that the index is based on self-reports of behaviour with the obvious limitations [36].

#### Category 5: index on functional ability

We identified one index that includes variables of functional ability, the Health Utilities Index (HUI) [32]. The HUI measures a child’s ability to function within eight health domains and combines these into a single health utility dimension [32]. The assessed variables are vision, hearing, speech, ambulation, dexterity, cognition, emotion and pain [32]. Each attribute is scored from 1 (representing full ability) to a maximum of 5 or 6 (representing poor functional ability) [32]. Using an existing algorithm based on population norms, the scores for the eight health domains were combined to produce a composite score that assigned a numeric utility value to the individual’s total health state, where 1.0 represented perfect health and 0.0 represented a health state equal to death [32]. Negative utility values are possible on the HUI, representing health states that are considered worse than death [32]. Face validity, consistency and sensitivity of the index are judged as good [56], however, consistency limited due to external evaluation by parents [32].

#### Category 6: index combining prenatal, postnatal and behavioural aspects

We identified one index attributed as a combination of different aspects, the Health Indicators for Swedish children [1, 33]. A composite index is built by combining information on children injured by external causes, exposed to tobacco in the womb, with low birthweight, that are breastfed, vaccinated against MPR (measles, parotitis and rubella) as well as information on teenage abortions [33]. The results for each indicator are added and the sum is divided by six, which results in an index between 0 and 100 with 100 representing the best score [33]. The index is described as a consistent and sensitive index, but the face validity is not provided [33].

## Discussion

With this scoping review, we provided an overview of the literature that report health indices on health outcomes and risk factors as well as health behaviour in children and adolescents. We identified and described these health indices and conducted quality appraisals of these indices.

In total, we identified 36 eligible studies and 18 health indices. The majority of the identified indices covered anthropometrical or dental variables. Furthermore, there were indices for newborns as well as indices assessing lifestyle and functional ability. One identified index combines different aspects out of all.

Interestingly, the number of indices identified was very limited.

Not all existing indicators for children and adolescents have been captured by our scoping review since these did not meet study’s inclusion criteria of a composite index. However, none of the provided composite indices gives a complete picture of child and adolescent health. To function as effective tools for evaluating and monitoring health, this complex “state of complete physical, mental and social well-being” other essential indicators or sub-indices should be incorporated or added [1, 57].

Indeed, in our review, composite indices did not include many health determinants except in two indices (Healthy Lifestyle Index (HLI); Health Indicators for Swedish Children), despite of their importance for health monitoring [4, 26].

Also, the topic of injuries, a relevant area in child health [4] and leading cause of death, disability and burden in children, could only be found in one index parameter (children injured by external causes in the index Health Indicators for Swedish Children). However, several publications identified through our search reported on single indicators related to these topics (for examples cited in this review see the work by Peoples-Sheps et al. and Kohen et al. [32, 40]). However not covered by our search results, indices on child road safety have been published, such as the work by Gitelman et al. that describes the development of a composite index of child road safety in a municipality [58]. In addition, the European Child Safety Alliance developed child safety report cards as tools that were specifically designed to bring attention to child injury as a major public health issue [59]. They include single indicators and composite indices [59].

Although the scoping review focused on children and adolescents, some of the indices were not limited for use in this age group and might have therefore been excluded. It is noteworthy, that in most studies indices were calculated with primary data collection. This is an important aspect when evaluating their applicability in monitoring and evaluation of health prevention and promotion initiatives where routine data use would most desirable for feasibility reasons.

Most of the indices identified in this review consist of anthropometrical variables and were used to identify obesity in the studied population. Both generalized and abdominal obesity are associated with increased risk of morbidity and mortality [52]. The main cause of obesity-related deaths is cardiovascular disease, for which abdominal obesity is a predisposing factor [52]. BMI is the predominantly chosen indicator to measure body size and composition, and to diagnose underweight and overweight [52] as easy to measure and calculate tool [48]. However, alternative measures that reflect abdominal adiposity, such as waist circumference, waist-to-hip ratio and waist-to-height ratio, have been suggested as being superior to BMI in predicting cardiovascular disease risk [52]. This is based largely on the rationale that increased visceral adipose tissue is associated with a range of metabolic abnormalities that are risk factors for type 2 diabetes and cardiovascular diseases [52]. Since the waist circumference depends on the percentage of body fat in the abdomen and in children and adolescents growth influences this value, the waist-to-height ratio is often formed in this age group to increase the significance [52].

For assessing newborns´ health, the Apgar score was reported. It remains a widely accepted method of assessment and is endorsed by both the American College of Obstetricians and Gynecologists and the American Academy of Pediatrics [54]. Apgar scores may vary with gestational age, birth weight, maternal medications, drug use or anaesthesia, and congenital anomalies [54]. Several components of the score are also subjective and prone to inter-rater variability [54]. Thus, the Apgar score is limited in that it provides somewhat subjective information about an infant’s physiology at a point in time [54]. The perinatal health index predicted childhood health correctly for 79% [27] of the studied children. However the value of perinatal health data in predicting subsequent morbidity was questioned by Gissler et al. [27] and the index turned out to be more precise for predicting health [27].

We included indices with dental variables in this scoping review as an important aspect of health, since “oral health affects people physically and psychologically and influences how they grow, enjoy life, look, speak, chew, taste food and socialize, as well as their feelings of social well-being” [41].

There are difficulties and shortcomings in measuring children’s and adolescents´ health with indices when there are too few and too incomplete indicators [33]. In many cases, data are insufficient to describe the broad and complex area that children’s and adolescents´ health represents [33]. There is a lack in the availability of routine data [33] which can also be seen in this scoping review, since most of the studies identified reports on primary data collection. Besides, the list of composite indices that emerged from this review reflects the lack of availability of appropriate indicators and indices in some essential dimensions of child health and well-being in general. Even though we searched the database PsycINFO, we were unable to identify indices on mental health. In another study from Sweden as well as in other publications [4, 5, 33, 57, 60], a lack of data on children’s mental health is described as well. Furthermore, it is reported that information about children’s physical environment and its impact on their health is stated and reliable data on cultural offerings and experiences are also very poor or lacking [4, 5, 33, 61]. Other missing indicators and topics have been stressed in numerous indicators projects in the past, such as intentional injuries & child abuse [4, 5, 57, 61], children’s social development [5], social capital and its influence on child health and development [33, 61], educational development & school social environment [4, 5, 62], biological determinants of child obesity [63] or the consideration of health promotion resources [64], amongst others. Thus, data availability is a central aspect to be considered when developing indicators and indices to monitor child’s health [33, 61] and must be taken into account, as has been done in other indicators’ selection processes [4, 29, 63]. Furthermore, the most valuable insights are provided by repeatedly measured indicators rather than single surveys [1, 61] . Routinely conducted, continuous monitoring can inform about changes over time, allowing to identify some emerging issues or to monitor the effect of interventions [5, 61]. Thus, the long-term availability of data remains an important prerequisite to the use of indicators and indices [65].

In some countries, health monitoring systems can rely on solid, widely available, and accessible health data [61]. In Germany, though, the problem of data availability in the field of health promotion monitoring and reporting still challenges researchers [66], especially on a local level, where more attention is needed [67, 68]. Promising developments to address this issue are underway [65, 67].

### Strengths and limitations

Limitations of our study were a) a broad study question with the potential to miss out certain papers because of the focus of the question on the presented PIC scheme with corresponding inclusion and exclusion criteria and key words in the search strategy, b) the selection and limitation to the reported databases with an emphasis on medical and/or health outputs and the potential to miss out certain reports or health reporting documents from other resources, i.e. grey literature or resources covering social sciences manuscripts, c) the acceptance of various formats of scientific literature with the limitation that the granularity of information differed according to the article format while d) some information such as quality appraisal information had to be retrieved through additional sources or were limited in scope and depth.

These limitations could have impacted our findings by various ways: we could have missed out certain indices and indicators that were not identified through the search strategy, included in papers that were not listed in the selected databases or excluded by us within the selection process. These could have mainly affected indices and indicators that cover adjacent areas such as social, environmental or educational topics that are undoubtedly very important for child health determination, but were not in the focus of this project. This might have led to an overemphasis of a “medical” or health view on our work.

In the future, this could be tackled by an even broader and deeper approach and scope of the work including additional key words and additional database searches such as like CINAHL and multidisciplinary databases like Emerald. This would most likely lead to the identification of further categories in additional areas exceeding the six we named.

A rigorous expert consensus procedure such as a Delphi or focus group that goes beyond our literature quality appraisal would strengthen the impact and a priority setting of the mentioned indices and indicators. This would be helpful for the implementation in practice, i.e. in the surveillance and monitoring of child health projects.

## Conclusion

In conclusion, this scoping review shows that indices may be pragmatic tools for monitoring and evaluating the health of children and adolescents, as well as to evaluate interventions in prevention and health promotion settings over time or between different groups [33]. Most indices identified were valid, consistent and sensitive. However, most indices would require primary data acquisition as prerequisite. Therefore, sets of single indicators of routine data will still have their role in monitoring children’s and adolescents´ health for the evaluation of preventive interventions. Easily available routine data of solid quality are necessary, both for single indicators and composite indices, to be able to provide relevant and effective tools to evaluate and monitor children’s and adolescents’ health in a society. A sensible selection of appropriate index and individual indicators adapted to given settings should be therefore made in any project through further reduction procedures (such as expert consensus).

## Supplementary Information

**Additional file 1: Supplementary file 1.** Search strategy for MEDLINE, EMBASE and PsycINFO.

## Data Availability

The publications assessed in the current study for eligibility (fulltext stage) are available in the OSF repository under the link (https://osf.io/nc5mt). Datasets used and/or analysed during the current study are available from the corresponding author on reasonable request.

## Abbreviations

PRISMA-ScR: PRISMA Extension for Scoping Reviews
WHO: World Health Organization
SDGs: Sustainable Development Goals
OECD: Organisation for Economic Co-operation and Development
CHILD: Indicators of Life and Development
HPM: Community Health Monitoring Programme
BMI: Body mass index
HLI: Healthy Lifestyle Index
MAMC: Mid-arm muscle circumference
DMFS: Decayed, missing and filled surfaces
DMFT: Decayed, missing and filled teeth
SiC index: Significant Caries Index
OHI(S): Oral Hygiene Index (simplified)
HUI: Health Utilities Index
