# Supplementary material for: Health indices for the evaluation and monitoring of health in children and adolescents in prevention and health promotion: a scoping review
Source: BMC Public Health. 2021 Dec 20;21:2309. doi: 10.1186/s12889-021-12335-x (PMC8686583; doi:10.1186/s12889-021-12335-x)
Supplement: Supplementary file 1 — Additional file 1: Supplementary file 1. Search strategy for MEDLINE, EMBASE and PsycINFO. [file 12889_2021_12335_MOESM1_ESM.docx]

## **Supplementary file 1. Search strategy for MEDLINE, EMBASE and PsycINFO**

Medline via Ovid

Database(s): **Ovid MEDLINE(R)**1946 to May Week 3 2020
Search Strategy: 26.05.2020

| **#** | **Searches** | **Results** |
| --- | --- | --- |
| 1 | child.ti,ab. | 287552 |
| 2 | children.ti,ab. | 908040 |
| 3 | teenager*.ti,ab. | 12601 |
| 4 | youth.ti,ab. | 52230 |
| 5 | adolescen*.ti,ab. | 233841 |
| 6 | 1 or 2 or 3 or 4 or 5 | 1233594 |
| 7 | (health adj3 index*).ti,ab. | 5288 |
| 8 | (health adj3 indices).ti,ab. | 1469 |
| 9 | (health adj3 indicator*).ti,ab. | 9230 |
| 10 | 7 or 8 or 9 | 15683 |
| 11 | "health promotion".ti,ab. | 25174 |
| 12 | prevention.ti,ab. | 463879 |
| 13 | monitoring.ti,ab. | 409889 |
| 14 | reporting.ti,ab. | 148891 |
| 15 | public health surveillance/ | 3418 |
| 16 | population surveillance/ | 58761 |
| 17 | 11 or 12 or 13 or 14 or 15 or 16 | 1060021 |
| 18 | 6 and 10 and 17 | 533 |
| 19 | (english or german).lg. | 22980514 |
| 20 | 18 and 19 | 481 |

Embase via Ovid

Database(s): **Embase**1974 to 2020 Week 21
Search Strategy: 26.05.2020

| **#** | **Searches** | **Results** |
| --- | --- | --- |
| 1 | child.ti,ab. | 418873 |
| 2 | children.ti,ab. | 1276908 |
| 3 | teenager*.ti,ab. | 19719 |
| 4 | youth.ti,ab. | 78800 |
| 5 | adolescen*.ti,ab. | 355806 |
| 6 | 1 or 2 or 3 or 4 or 5 | 1739125 |
| 7 | (health adj3 index*).ti,ab. | 8790 |
| 8 | (health adj3 indices).ti,ab. | 2097 |
| 9 | (health adj3 indicator*).ti,ab. | 12763 |
| 10 | 7 or 8 or 9 | 23224 |
| 11 | "health promotion".ti,ab. | 34382 |
| 12 | prevention.ti,ab. | 688035 |
| 13 | monitoring.ti,ab. | 676659 |
| 14 | reporting.ti,ab. | 253017 |
| 15 | public health surveillance/ | 172541 |
| 16 | population surveillance/ | 174305 |
| 17 | 11 or 12 or 13 or 14 or 15 or 16 | 1743142 |
| 18 | 6 and 10 and 17 | 1169 |
| 19 | (english or german).lg. | 30215879 |
| 20 | 18 and 19 | 1049 |

PsycInfo via EBSCO

Database(s): **APA** **PsycInfo**
Search Strategy: 26.05.2020

| **#** | **Searches** | **Results** |
| --- | --- | --- |
| S20 | S18 AND S19 | 116 |
| S19 | LA (english OR german) | 3,228,353 |
| S18 | S6 AND S10 AND S17 | 118 |
| S17 | S11 OR S12 OR S13 S14 OR S15 OR S16 | 96,063 |
| S16 | MA "population surveillance" | 4,242 |
| S15 | MA "public health surveillance" | 68 |
| S14 | TI reporting OR AB reporting | 44,435 |
| S13 | TI monitoring OR AB monitoring | 43,865 |
| S12 | TI prevention OR AB prevention | 83,094 |
| S11 | TI "health promotion" OR AB "health promotion" | 10,415 |
| S10 | S7 OR S8 OR S9 | 5,582 |
| S9 | TI (health N3 indicator*) OR AB (health N3 indicator*) | 3,356 |
| S8 | TI (health N3 indices) OR AB (health N3 indices) | 2,257 |
| S7 | TI (health N3 index*) OR AB (health N3 index*) | 1,838 |
| S6 | S1 OR S2 OR S3 OR S4 OR S5 | 530,712 |
| S5 | TI adolescen* OR AB adolescen* | 155,979 |
| S4 | TI youth OR AB youth | 66,249 |
| S3 | TI teenager* OR AB teenager* | 5,732 |
| S2 | TI children OR AB children | 401,564 |
| S1 | TI child OR AB child | 401,564 |

## 
